# Forest Topsoil Organic Carbon Declines Under Ash Dieback

**DOI:** 10.1111/gcb.70430

**Published:** 2025-08-19

**Authors:** Fiona M. Seaton, David A. Robinson, Claire M. Wood, Clare M. H. Benskin, Rebecca L. Rowe, Karen Hornigold, Keith J. Kirby, Chris Nichols, Simon M. Smart

**Affiliations:** ^1^ UK Centre for Ecology & Hydrology Lancaster UK; ^2^ UK Centre for Ecology & Hydrology Environment Centre Wales Bangor UK; ^3^ Lancaster University Lancaster Environment Centre Lancaster UK; ^4^ Woodland Trust Grantham UK; ^5^ Department of Biology Oxford UK

**Keywords:** *Fraxinus excelsior*, *Hymenoscyphus fraxineus*, loss on ignition, soil organic matter, soil pH, tree disease, woodland

## Abstract

Tree diseases are increasingly affecting woodland ecosystems across the world. However, the impact of these diseases upon the soil, and in particular soil carbon, is still poorly understood. Here we present the results of a field survey of ~100 woodlands across Great Britain measured in 1971, 2001 and 2022 and evaluate the fifty‐year trend in topsoil (0–15 cm) carbon based upon measurements of soil organic matter (SOM) and the impact of *Hymenoscyphus fraxineus* (ash dieback). To better represent the full SOM distribution, including the extremely high SOM measurements, we adopt a Beta mixture modelling approach within a Bayesian framework. Across all woodlands, comprising ~1,500 plots per survey, average SOM remained constant across the fifty‐year time series. However, the 311 plots with ash dieback had lower SOM in the most recent survey compared to the 328 plots with ash trees present but no dieback recorded, due to a slight decline in SOM under ash dieback. This resulted in plots with ash dieback having a modelled mean SOM of 12.2% compared to 13.4% in plots without ash dieback, a difference of 1.23 percentage points (95% CI 0.25–2.21). Ash dieback was more likely to be recorded in plots that had higher soil pH pre‐ash dieback invasion, but the decline in SOM under ash dieback was not explained by changes in soil pH or changes in the ground flora composition. Converting our results to soil C and extrapolating for broadleaved woodland across the entirety of Great Britain, the total amount of topsoil carbon lost to date due to ash dieback could be 6 MtCO_2_ (± 4 s.d.). Our results show the importance of understanding the impacts of tree disease when considering current and future woodland carbon dynamics.

## Graphical abstract

Despite the rapid expansion of tree diseases across the world, there is still limited knowledge on how diseases such as ash dieback will impact carbon cycling, especially belowground. We use data from a nationally representative survey of British broadleaved woodlands covering 50 years of change and find that soil carbon has declined under ash dieback. This raises concerns about the long‐term resilience of forest soil carbon stocks and soil health due to the increasing levels of forest disturbance.

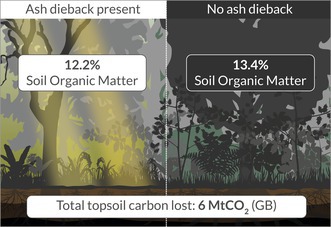

## 1 Introduction

Forests are increasingly under threat globally from a variety of different stressors, including climate change, deforestation, storms, wildfires, changes in management, insect infestations and tree disease (Millar and Stephenson 2015). Tree disease, in particular, is increasing in prevalence and severity globally, with exponential growth in invasive forest pathogens in the past few decades (Gougherty 2023; Santini et al. 2013). Other types of disturbance to woodland ecosystems are known to decrease soil organic matter (SOM) and the associated soil carbon stocks, with effects that can persist for decades (Mayer et al. 2020, 2024). However, the impacts of tree disease upon soil dynamics are currently poorly understood (Holden and Treseder 2013; Mayer et al. 2024; Zhang et al. 2015). If widespread tree diseases such as ash dieback (*Hymenoscyphus fraxineus*) have similar effects upon soil carbon as other disturbance types, there could be considerable implications for forest carbon stocks globally. This is particularly relevant as carbon stored in forest soil accounts for around half of all forest carbon stocks (Pan et al. 2011). Therefore, we need to better understand and quantify the impact of tree diseases upon the soil.

Ash dieback has been widespread across Europe for several decades, leading to considerable mortality of ash ( *Fraxinus excelsior* ) and further consequences for the wide variety of species that rely on ash (Combes et al. 2024). Ash dieback could impact SOM and soil carbon through a variety of mechanisms, either through the short‐term impacts of dieback such as defoliation and canopy opening or through the replacement of ash within the tree community. Ash has distinct effects upon the soil, with shallower rooting depth, a greater ratio of fine root biomass and a higher nutrient profile in both litter and root exudates compared to other tree species (Mitchell et al. 2014; Staszel‐Szlachta et al. 2024; Webb et al. 2022). In general, ash therefore appears more often on higher pH and lower soil carbon soils than other common European broadleaved trees (Mitchell et al. 2021), with common garden experiments showing that ash trees increase soil pH and decrease carbon (Staszel‐Szlachta et al. 2024). Therefore, the loss of ash from European woodlands might be expected to lead to an increase in soil carbon and decrease in pH as other tree species take its place. However, forest disturbance and the opening of gaps in the canopy, through biotic or management activities, usually lead to a loss in soil carbon (Mayer et al. 2020, 2024; Tong et al. 2024). The short‐term impacts of ash dieback, with increased litter inputs due to defoliation, loss of root‐derived inputs, opening of the canopy and shifts in soil hydrology, could lead to changes in soil microbial activity that decrease the soil carbon content (Dahlsjö and Malhi 2024; Gómez‐Aparicio et al. 2022). Observed shifts in the ground flora composition following ash dieback could also lead to changes in nutrient cycling and decomposition rates (Brunet et al. 2023; Seaton et al. 2025). The impacts of ash dieback also occur against a backdrop of great change in woodland soils, driven by factors such as the dramatic decline in acid rain which has led to a large increase in soil pH since the 1970s (Seaton et al. 2023).

Understanding the impacts of ash dieback upon soil organic matter (SOM) requires long‐term woodland soil data, as woodlands respond to disturbances and global threats over longer than decadal timeframes (Rackham 2008). Here, we use data from the ‘Bunce’ woodland survey, a long‐term survey of established woodlands across Great Britain, to evaluate the impact of ash dieback using data from before and after the spread of ash dieback across the UK (Smart, Walker, et al., 2024; C. M. Wood et al. 2015). Within this survey, in 1971, 2001 and 2022, samples of the topsoil (0‐15 cm) were analysed for SOM and soil pH. Within the most recent survey, the presence of ash dieback was also recorded by the surveyors, providing a unique opportunity to evaluate the impacts of ash dieback on SOM. Previous analysis of the survey data has shown that ground flora composition shifted towards a more diverse, forb‐dominated community under ash dieback; however, the impacts of ash dieback on SOM and soil pH are as yet unknown (Seaton et al. 2025). Our hypotheses were: (1) overall topsoil organic matter would have remained relatively constant over the 50 years of survey; (2) the subset of our plots that had experienced ash dieback would have shown a decrease in SOM from 2001 to 2022; and (3) that at least part of this change in SOM would have been mediated by changes in soil acidity and the ground flora—represented by changes in forb cover.

## 2 Methods

### 2.1 Field Survey

The ‘Bunce’ field survey first took place in 1971, with two follow‐up surveys completed in 2000–2003 and 2020–2022 (henceforth referred to as the 2001 and 2022 surveys) respectively. In the 1971 survey, 103 broadleaved woodland sites were visited; sites were selected to be representative of the range of broadleaved woodland plant species composition across Great Britain. This was done by multivariate clustering of a previous, more comprehensive survey of 2453 British woodlands. This produced 103 woodland types, from each of which a single representative site was chosen. The resulting sites are spread across environmental zones in a similar ratio to that of the total amount of broadleaved woodland in Great Britain. Around two‐thirds of the sites were subsequently identified as ancient woodland. Sites ranged in size from 4 to 100 ha, with a single outlier of 312 ha, with an overall mean size of 31.8 ha.

Within each site, 16 plots measuring 14.1 × 14.1 m were surveyed for vegetation and a soil core taken. In the resurveys, the same sites and plots were revisited and resurveyed using the same methods as far as possible, except that some plots are missing from the more recent surveys due to land access being denied or to them no longer being woodland. The average date of survey was also later in the year for the first survey, as due to logistical reasons, the entirety of the first survey took place from July onward, whereas the more recent surveys started in May. For further details on the survey, see C. M. Wood et al. (2015) and Smart, Walker, et al. (2024). In the most recent survey, the presence of ash dieback was recorded by the surveyors, with a total of 311 plots containing evidence of ash dieback (21% of all plots) and 328 plots containing ash trees but no evidence of ash dieback (22% of all plots). There were also 849 plots that had no ash, of which 678 were in sites where ash was present in another plot, indicating that ash could survive in the wider site conditions. Compared to plots with ash present but no dieback, plots with ash dieback present had, on average, more dead trees (10% of all trees were dead compared to 5%, driven by 18% of ash trees being dead compared to 7% in non‐dieback plots). Few plots showed evidence of recent extractive management (< 5% of plots either with or without dieback).

### 2.2 Soil Methods

A soil core down to 15 cm depth was taken in a central location in every plot. In the first two surveys, these were taken using a trowel and approximately 1 kg of soil was taken, while in the most recent survey a 5‐cm‐diameter plastic tube was used to extract a soil core. The loose litter layer is first removed, but the fermentation and humus layers were sampled. In the first survey, a small pit and augur sampling were used to examine the deeper soil horizons and assign each of the plots to a soil group according to the Avery (1973) classification. A count of the number of plots within each soil group for the entire survey as well as the subset of plots with ash or ash dieback present is available in Table S1. Soil pH of the fresh soil in deionised water (1:1) was measured. Soil organic matter was measured through loss on ignition (LOI) analysis after air‐drying and sieving, followed by combustion at 550°C for 6 h (2 h in the 1971 and 2001 surveys). For the later carbon stock change calculation, SOM was converted to soil organic carbon (SOC) concentration by multiplying by 0.55 (Emmett et al. 2010). Efforts to keep the methodologies consistent across all three surveys were taken, with a program of remeasurement and cross‐validation undertaken in the 2001 survey due to changes in technology; for further details, see C. M. Wood et al. (2015).

### 2.3 Statistical Analysis

Soil organic matter was modelled by fitting a Beta mixture model using a Bayesian approach in Stan (Carpenter et al. 2017; Stan Development Team 2024). There were two Beta distributions included within the model, each with its own intercept and shape parameter. The probability of each observation corresponding to the mixture components (i.e., the mixing proportion) is modelled with the $\theta_{k}$ parameter, which is a simplex (i.e., sums to one). The intercept (α) was constrained to be positive ordered and the shape parameter (ϕ) constrained to be negative ordered. This corresponds to assuming the first mixture component has a lower mean and a narrower peak than the second mixture component. The Beta distributions were modelled using the mean ($\mu_{k}$) and shape ($\phi_{k}$) parameterisation, the equation is given below for k mixture components, i observations, j fixed effects and l hierarchical effects:

$$p_{Y}\left(y|\theta ,\mu ,\phi \right)=\sum_{k=1}^{K}\theta_{k}\text{Beta}\left(y|a_{k},b_{k}\right),\;a_{k}=\mu_{k}\phi_{k},\;b_{k}=\left(1-\mu_{k}\right)\phi_{k}$$

$$\text{logit}\left(\mu_{k}\right)=\alpha_{k}+\sum_{l=1}^{L}\alpha_{k,l}+\beta_{j,k}*X_{j}$$

$$\alpha_{k,l}∼N\left(0,\sigma_{k,l}\right)$$

where $\alpha_{1}<\alpha_{2}<\ldots <\alpha_{k}$ and $\phi_{1}>\phi_{2}>\ldots >\phi_{k}$. Within the hierarchical effects the two σ parameters vary by mixture component but the individual group‐level estimates are assumed to be constant across the different mixture components. The fixed effects differed for each model, with the change over time model including only the survey year, and the ash dieback model including an interaction between survey year and ash dieback status. The potential impacts of mediating effects were tested in a model that included an interaction between survey year and ash dieback status, as well as direct effects of pH (chosen due to the importance of pH in soil dynamics), forb cover (chosen due to the known increase in forb cover under ash dieback) and day of year (chosen to test the possibility that differences between survey timing between the first and subsequent surveys could be affecting our results). The full Stan model code is available in the Supporting Information. Soil pH was modelled as a Gaussian response to survey year and ash dieback using the brms package (Bürkner 2017).

All models were run with 4 chains, each with 4000 iterations (half warm‐up); Rhat was confirmed to be below 1.01 and effective sample size above 1000 for all model parameters. Graphical posterior retrodictive checks were performed to confirm a good fit to the data. Models of the impact of ash dieback upon SOM were trained only on plots that contained ash and/or ash dieback in the 2022 survey, resulting in 632 plots included with 1896 measurements in total across the three surveys. Contrasts between years and/or ash dieback treatments were calculated by taking the difference between predictions when every datapoint within the original data was predicted under each contrast. This is equivalent to the method used within the emmeans package, which was used for the soil pH model results (Lenth 2024).

### 2.4 Soil Carbon Stock Change Calculation

Estimated change in topsoil carbon stock across British woodlands resulting from ash dieback was calculated based upon an estimation of the affected area of woodland in Great Britain, the volume and mass of soil impacts based on a calculation of bulk density drawn from the Countryside Survey data and the modelled change in soil C predicted by the Beta mixture model (described above) from 2001 to 2022 with a conversion of SOM to SOC, as described below.

Bulk density of the fine earth (< 2 mm) was calculated based upon applying a pedotransfer function to the mean SOM values returned by the Beta mixture model. Due to the known underestimation of bulk density in woodland soils based on currently available pedotransfer functions (Panagos et al. 2024), we used broadleaved woodland data collected as part of the Countryside Survey (CS) to create our models to estimate bulk density (Emmett et al. 2010). Survey methods were consistent between the Bunce survey and CS. In total, 94 woodland plots from 71 1 km CS squares were used for these pedotransfer models; all models were fitted using a generalised additive mixed effect modelling approach using the mgcv R package (S. N. Wood 2004) with a random effect of the 1 km square upon the intercept. Data from the CS 2007 survey were used to fit a model of bulk density as a smooth function of SOM; this was used to estimate the 2001 bulk density values for our survey (Figure S1a). The change in bulk density from survey to survey was predicted based upon both the initial SOM value and the change in SOM from the 2007 to the 2018–22 CS surveys (Figure S1b). These predicted bulk density values were used to calculate the total weight of the topsoil (down to 15 cm), which was used to calculate loss values per ha based on the model estimated carbon lost per kg of soil. We used all draws from the model estimated SOM loss in ash dieback plots from 2001 to 2022 and converted the SOM to soil carbon concentration by multiplying by 0.55 (Emmett et al. 2010). The loss of CO_2_ was calculated by multiplying carbon stock values by 3.67.

To extrapolate across Great Britain, the total area of broadleaved woodland was taken from the Land Cover Map 2015 (Rowland et al. 2017). The area of broadleaved woodland was then multiplied by the proportion of plots within the Bunce woodland sites that had ash dieback present (21%) to get the total area of woodland that we consider to be equivalent to our ‘ash dieback present’ category. For creation of the uncertainty simulations, this proportion of area was allowed to vary with a normal distribution on the logit scale, which gave a distribution ranging from 10% to 40% of the area with the interquartile range being from 18% to 24%. 8 thousand simulations were run with a reasonable set of parameter estimates to give an estimate of the uncertainty of our estimated change in carbon stock.

## 3 Results

### 3.1 Change Over Time in Woodland SOM

**FIGURE 1:**
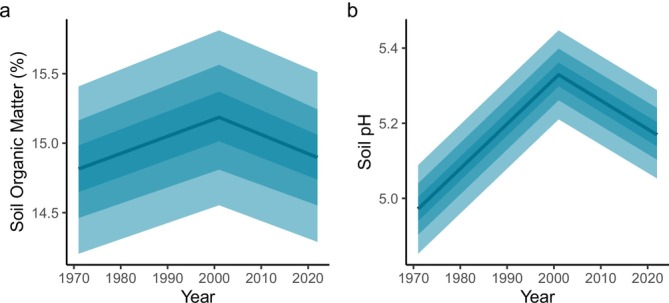
Mean SOM (a) and soil pH (b) and the uncertainty in that mean over time for all woodland plots. The central blue line shows the median model estimated mean, while the shaded blue ribbons show the 25%, 50% and 75% confidence intervals of the estimated mean.

Our woodlands showed a wide range in soil properties, with SOM ranging from 0.8% to 97.5% (interquartile range 9.5% to 20.1%) and pH ranging from 2.9 to 10.0 (interquartile range 4.2 to 5.9). Across all woodlands surveyed, model estimated mean SOM remained constant between the three surveys (Figure 1a). While the model showed a slight increase in average SOM from 1971 to 2001 from 14.81% to 15.19%, then a decrease in 2022 to 14.90%, both the 95% and 80% CI of these changes overlapped with zero, indicating an overall stable trend over time (Figure 1a and Table 2). Within the Beta mixture model, the majority of the SOM measurements (80%) were associated with the mineral soils region, with a smaller number of measurements associated with a long tail within the more organic soil region (Table 1 and Figure S2). Average soil pH increased from 4.97 to 5.33 from 1971 to 2001 and then declined in 2022 to pH 5.17, intermediate between the 1971 and the 2001 averages (Figure 1b and Table 2).

**TABLE 1:** Estimated values for the parameters common to all SOM models, comprising the models of change over time (Total Change), ash dieback effects (Ash Dieback) and estimated impacts of potential mediating variables (Mediation). Each parameter is given as the mean estimate (± standard deviation). Parameters included here are theta (θ – proportio*n* of data points within mixture component one), plus for each mixture component the intercept, phi (ϕ – a shape parameter for the Beta distribution), and sigma parameters (σ – standard deviation of the site and plot‐level hierarchical effect).

Parameter	Mixture component	Model		
		Total change	Ash dieback	Mediation
theta	—	0.80 (± 0.01)	0.83 (± 0.02)	0.83 (± 0.02)
intercept	One	−1.90 (± 0.03)	−1.91 (± 0.04)	−1.91 (± 0.04)
phi	One	86.9 (± 4.61)	123.7 (± 10.3)	121.3 (± 10.1)
sigma (site)	One	0.31 (± 0.02)	0.30 (± 0.02)	0.29 (± 0.02)
sigma (plot)	One	0.20 (± 0.01)	0.20 (± 0.02)	0.20 (± 0.02)
intercept	Two	−0.60 (± 0.07)	−1.04 (± 0.10)	−1.04 (± 0.10)
phi	Two	3.27 (± 0.18)	5.75 (± 0.57)	5.87 (± 0.63)
sigma (site)	Two	0.39 (± 0.04)	0.33 (± 0.05)	0.35 (± 0.05)
sigma (plot)	Two	0.03 (± 0.03)	0.05 (± 0.04)	0.05 (± 0.04)

**TABLE 2:** Differences in mean SOM and pH between the different survey periods, given as the model‐estimated mean change (later year minus earlier year, i.e., positive values represent an increase), and the 80% and 95% confidence intervals of that change.

Variable	Time period	Mean	80% CI	95% CI
SOM	1971 to 2001	0.37	−0.17 to 0.91	−0.42 to 1.18
SOM	2001 to 2022	−0.29	−0.85 to 0.27	−1.15 to 0.57
SOM	1971 to 2022	0.08	−0.43 to 0.59	−0.72 to 0.89
pH	1971 to 2001	0.36	0.39 to 0.33	0.41 to 0.31
pH	2001 to 2022	−0.16	−0.12 to −0.19	−0.11 to −0.21
pH	1971 to 2022	0.20	0.23 to 0.17	0.25 to 0.15

### 3.2 Ash Dieback Effects

**FIGURE 2:**
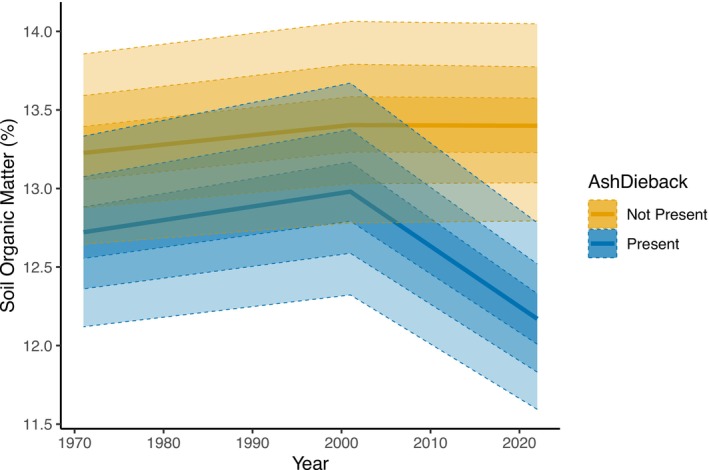
Change in SOM over time in ash plots with (blue) and without (yellow) ash dieback. The central line shows the median model estimated mean, while the shaded ribbons show the 25%, 50% and 75% confidence intervals of the estimated mean.

Plots with evidence of ash dieback declined in SOM between 2001 and 2022, whereas ash plots without ash dieback showed no change in SOM over the same time period (Figure 2). Note that the average SOM shown in Figure 2 is lower than the average SOM across all plots shown in Figure 1, with an average SOM of ~13% in plots with ash present compared to ~15% within plots without ash. There was no difference found between the plots with and without ash dieback in 1971 and 2001 (Table 3). However, in 2022, plots with ash but without dieback had a higher average SOM of 13.41% compared to the 12.18% SOM with ash dieback, and the 95% CI of this did not overlap zero (Table 3). The decrease from 2001 to 2022 in mean SOM under ash dieback was estimated as −0.81 percentage points; however, despite SOM being lower under ash dieback in the most recent survey, the 95% CI of the change between surveys did overlap zero (−1.79 to 0.17). This decrease under ash dieback was, however, the only change between the different survey years where the 80% CI did not overlap zero (−1.45 to −0.17). The Beta mixture model was similar in its fit to the data and parameter estimates as within the change over time model described above. The only notable differences from the change over time model were changes in the phi and sigma parameters, which represent the ash dieback effects accounting for variability that was incorporated into the variance and hierarchical effects in the change over time model (Table 1). The effect of ash dieback upon SOM occurred only within the first mixture component, which corresponds to the more mineral soils (Figure S3), which showed a 1.04 percentage point lower mean SOM in plots with ash dieback compared to plots without ash dieback (95% CI is 0.29–1.78).

**TABLE 3:** Estimated difference in SOM between plots without ash dieback and plots with ash dieback given in percentage points for models with and without mediating variables.

	Model without covariates					Model with covariates				
		Quantiles					Quantiles			
Year	Estimate	2.5%	10%	90%	97.5%	Estimate	2.5%	10%	90%	97.5%
1971	0.520	−0.390	−0.070	1.090	1.410	0.530	−0.330	−0.030	1.100	1.440
2001	0.430	−0.610	−0.250	1.110	1.460	0.440	−0.590	−0.240	1.120	1.470
2022	1.230	0.250	0.610	1.860	2.210	1.240	0.290	0.620	1.870	2.220

### 3.3 Potential Mediating Variables

**FIGURE 3:**
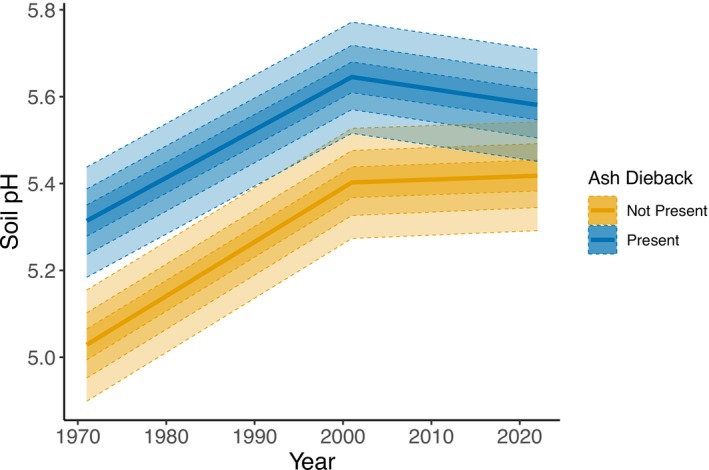
Change in soil pH over time in ash plots with (blue) and without (yellow) ash dieback. The central line shows the median model estimated mean, while the shaded ribbons show the 25%, 50% and 75% confidence intervals of the estimated mean.

At all time points, soil pH was higher in plots that had ash dieback versus those that did not (Figure 3) (difference in 1971 0.29 (95% CI 0.13–0.44); difference in 2001 0.24 (95% CI 0.08–0.40)), although the magnitude of this difference was reduced in the most recent survey (0.16 (95% CI 0.00–0.32)) (Figure 3).

**FIGURE 4:**
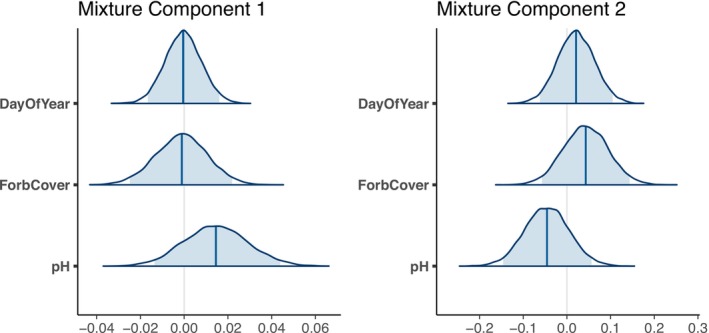
Estimated model coefficients for the two mixture components, the inner 95% interval is shaded light blue and the median shown as a dark blue line.

The effect of ash dieback upon SOM persisted even after accounting for changes in soil pH, day of year of survey and forb cover (Table 3). Of the identified potential mediating variables, only soil pH was found to show some potential relation to soil organic matter (Figure 4) although the 95% CI crossed zero and the direction of the effect differed by mixture component. Within the model with covariates, the percentage of observations that were allocated to the first mixture component was similar to the ash dieback model without covariates, at 83%, as were the other model parameters (Table 1).

### 3.4 Carbon Stock Change

The loss of topsoil carbon stock within our plots with ash dieback was estimated to be −5.29 MgC ha^−1^ between 2001 and 2022 (80% range −9.46 to −0.98). If we assume that the patterns seen across our surveyed woodlands can be extrapolated across the broadleaved woodlands in Great Britain, the total topsoil carbon lost across Great Britain between 2001 and 2022 due to ash dieback can be estimated as −1.59 MtC (80% range −3 to −0.29). This is equivalent to −5.82 MtCO_2_ (80% range −11.01 to −1.08).

## 4 Discussion

While there is considerable uncertainty within our estimate of potential carbon lost due to ash dieback, the estimated value of −5.8 MtCO_2_ is in total equivalent to more than half the annual carbon currently sequestered by broadleaved forests across Great Britain (JNCC 2024). As the average year of first recorded ash dieback infection across all 10 km squares of Great Britain is 2016 (Forestry Commission 2025), and our average year of survey is 2021, this could represent an average rate of loss of around −0.32 MtC yr.^−1^ (−1.16 MtCO_2_ yr.^−1^). These results show how biotic disturbance can greatly impact soil carbon in woodlands, which is a Europe‐and Asia‐wide issue in the case of ash (Carroll and Boa 2024), and indicates how important it is to account for the increasing rates of pathogen spread when considering future woodland carbon dynamics. Our estimates are on a similar scale to that seen in the soil organic layer in response to other types of disturbance events such as tree harvesting or windstorms (Mayer et al. 2024). These results also do not account for any impacts on above‐ground carbon stored with the ash trees, the fate of which will depend on management undertaken, but could incur additional losses (Reay 2013). The extent of the disturbance caused by ash dieback may differ in future, as temperatures approach or even surpass the optimal growth temperature of 20°C and forests experience more drought stress, which could decrease mortality from dieback (Combes et al. 2024). The scale of these impacts may also increase as ash dieback spreads further across the country. Other types of disturbances, such as storm damage or insect pest invasion, could act synergistically with ash dieback and lead to greater disturbance to the woodland canopy and the rest of the woodland‐soil ecosystem (Seidl et al. 2017; Sun et al. 2024; Tong et al. 2024).

Within woodlands not affected by ash dieback, we have found patterns of constant topsoil organic carbon and increasing pH that are consistent with previous studies across Great Britain (Rivas Casado et al. 2022; Seaton et al. 2023). While other studies have shown an increase in woodland soil carbon, this has largely been in forest plantations or recently afforested areas (Chapman et al. 2013). The woodlands studied here are well established, and in two‐thirds of cases are categorised as ancient woodland, and so it is not surprising that the topsoil organic matter has remained largely in equilibrium over the past 50 years. In addition, the majority of our woodlands are not undergoing any form of extractive management, with increasing amounts of deadwood and much lower tree removal rates than were experienced prior to the late 20th century (Smart, Walker, et al. 2024). Harvesting of trees and management‐based disturbance is known to reduce soil C stocks (Mayer et al. 2020). However, our results do clearly show that disturbances such as ash dieback can threaten this equilibrium and lead to losses of soil organic matter.

The decline in soil organic matter under ash dieback represents the opposite trend that would be expected from the loss of ash trees and replacement with other species. Soils under ash generally show higher decomposition rates compared to other tree species, related to greater enzyme exudation by ash roots and highly labile ash litter (Staszel‐Szlachta et al. 2024). This results in a lower soil carbon under ash relative to other species (Mitchell et al. 2021), so it does not fit the hypothesis that the replacement of ash with other species could be causing this change. Also, in most woods, there has not been enough time for this replacement to happen. Instead, declines in SOM may be due to a wide variety of factors including defoliation and the opening of canopy gaps leading to increased solar irradiation, increased soil temperature and changes in soil moisture (De Frenne et al. 2021). While the precise effects of canopy gaps upon decomposition rates vary depending on the local conditions (e.g., Forrester et al. 2023; Lenk et al. 2024), a meta‐analysis suggests the average effect of canopy gaps results in greater decomposition of existing carbon in the soil (Tong et al. 2024). Changes in decomposition related to the opening of canopy gaps could decrease soil carbon through causing changes in the carbon content of the upper soil horizons or by decreasing the depth of the organic horizon. We found that the ash dieback effect occurred mostly within the more mineral topsoils, which potentially indicates that ash dieback is not just impacting the Of and Oh soil layers through changes in litter (de‐)composition but also deeper soil layers. The changes in topsoil we have found could be masking changes in soil carbon deeper within the soil, as different tree species are known to have different soil carbon and rooting profiles (Steffens et al. 2022; Webb et al. 2022). Whilst deeper soil layers are in general less responsive to disturbance events and contain a lower concentration of carbon, our sampling is shallow, and sampling of deeper soil layers would provide a more complete picture (Balesdent et al. 2018).

We found no difference in SOM pre‐dieback, but there were differences in soil pH, with dieback more likely to be recorded in plots that had slightly higher pH initially. This is consistent with previous results showing that crown defoliation is greater in ash trees within higher pH sites (Turczański et al. 2020). As ash is more prevalent on higher pH sites, this pH effect could be due to higher densities of ash trees leading to higher mortality rates (Combes et al. 2024). Ash mortality is also known to be higher in areas of higher soil moisture; however, in non‐riparian woodlands, pH is usually lower under high moisture conditions, so this is unlikely to explain the pH effect (Klesse et al. 2021; Slessarev et al. 2016). Acidity increased under ash dieback, although pH remained higher than plots without ash dieback present. The increase in acidity under ash dieback may be due to changes in the composition and depth of the litter layer due to defoliation, or changes in root enzyme exudation, and could relate to changes in the soil microbial community, which are known to respond to dieback events in other tree species (Ávila et al. 2021; Gómez‐Aparicio et al. 2022; Zhang et al. 2015).

The longer‐term effects of ash dieback may be poorly predicted by the impacts we have detected, as the loss of ash trees from woodlands will lead to a shift in the tree community composition with knock‐on effects upon the soil communities that are not yet apparent. Ash trees have distinct nutrient profiles and rooting characteristics compared to other European trees, with more easily decomposed litter, greater root exudation and greater fine root biomass (Mitchell et al. 2021; Staszel‐Szlachta et al. 2024; Webb et al. 2022). This means that the soils under ash trees often have greater nutrient input and hydraulic conductivity, leading to distinct microbial communities and higher microbial respiration. Therefore, the loss of ash from woodlands could actually lead to an increase in soil carbon in the longer term, if replaced by other tree species of equal productivity within those environmental conditions. However, this assumes that the woodlands reach a steady state again. In practice, woodlands are experiencing increasing rates of disturbance events from a variety of sources including pathogens, insects and extreme weather events; thus, global woodland soil carbon stocks are increasingly at risk of depletion (Rackham 2008; Seidl et al. 2017).

## 5 Concluding Statement

The long‐term consequences of ash dieback on woodland SOM remain uncertain, but we have clearly demonstrated that disease, such as ash dieback, can have significant short‐term to medium‐term negative impacts. These findings have implications for future climates and support our understanding of the risk tree diseases pose to policies and scenarios utilisation of forests for climate mitigation.

## Author Contributions

**Fiona M. Seaton:** conceptualization, data curation, formal analysis, investigation, methodology, software, visualization, writing – original draft, writing – review and editing. **David A. Robinson:** conceptualization, funding acquisition, project administration, supervision, writing – review and editing. **Claire M. Wood:** data curation, investigation, project administration, resources, writing – review and editing. **Clare M. H. Benskin:** investigation, methodology, resources, writing – review and editing. **Rebecca L. Rowe:** validation, writing – review and editing. **Karen Hornigold:** funding acquisition, project administration, writing – review and editing. **Keith J. Kirby:** conceptualization, methodology, writing – review and editing. **Chris Nichols:** funding acquisition, project administration, writing – review and editing. **Simon M. Smart:** conceptualization, data curation, funding acquisition, project administration, supervision, writing – review and editing.

## Conflicts of Interest

The authors declare no conflicts of interest.

## Supporting information


**Figure S1.** Data and model fits used to estimate bulk density as a function of LOI (a), and change in bulk density based on an interaction between change in LOI and the log of the original LOI (logLOI07) (b). The central line represents the mean estimate and the shaded ribbons the 95% CI. Model estimated change in bulk density (b) is shown for three example original LOI values, corresponding to ~5% (green), ~12% (orange), and ~29% (purple).
**Figure S2.** Density of actual data (black line) compared to model fit over all components (a, blue lines) and by mixture component (b). In (b) the model predicted densities are coloured by mixture components, with the high orange peak being the first mixture component and the lower blue lines the second mixture component.
**Figure S3.** Change over time in ash plots with (blue) and without (yellow) ash dieback for the first mixture component (left, 80% of data, representing the majority of mineral soil measurements) and the second mixture component (right, 20% of data, representing the tail of high SOM values). The central line shows the median model estimated mean, while the shaded ribbons show the 25%, 50% and 75% confidence intervals of the estimated mean.
**Table S1.** Number of plots within each soil group according to the classification of Avery (1973).

## Data Availability

All data is publicly available within the NERC Environmental Information Data Centre, with our woodland survey data being found under Kirby et al. (2013, DOI: 10.5285/fb1e474d‐456b‐42a9‐9a10‐a02c35af10d2) and Smart, Wood, et al. (2024, DOI: 10.5285/42c203c8‐44de‐40e2‐a694‐b1e8cbd4c8e1). The Countryside Survey data used to create the bulk density pedotransfer functions can be found under Emmett et al. (2016, DOI: 10.5285/79669141‐cde5‐49f0‐b24d‐f3c6a1a52db8), Reinsch et al. (2023, DOI: 10.5285/d53fdf1d‐767a‐4046‐821a‐ea645001ddd3), and Bentley et al. (2023, DOI: 10.5285/af6c4679‐99aa‐4352‐9f63‐af3bd7bc87a4).
