# Supplementary material for: Forest Topsoil Organic Carbon Declines Under Ash Dieback
Source: Glob Chang Biol. 2025 Aug 19;31(8):e70430. doi: 10.1111/gcb.70430 (PMC12364553; doi:10.1111/gcb.70430)
Supplement: Supplementary file 1 — Figure S1. Data and model fits used to estimate bulk density as a function of LOI (a), and change in bulk density based on an interaction between change in LOI and the log of the original LOI (logLOI07) (b). The central line represents the mean estimate and the shaded ribbons the 95% CI. Model estimated change in bulk density (b) is shown for three example original LOI values, corresponding to ~5% (green), ~12% (orange), and ~29% (purple). Figure S2. Density of actual data (black line) compared to model fit over all components (a, blue lines) and by mixture component (b). In (b) the model predicted densities are coloured by mixture components, with the high orange peak being the first mixture component and the lower blue lines the second mixture component. Figure S3. Change over time in ash plots with (blue) and without (yellow) ash dieback for the first mixture component (left, 80% of data, representing the majority of mineral soil measurements) and the second mixture component (right, 20% of data, representing the tail of high SOM values). The central line shows the median model estimated mean, while the shaded ribbons show the 25%, 50% and 75% confidence intervals of the estimated mean. Table S1. Number of plots within each soil group according to the classification of Avery (1973). [file GCB-31-e70430-s001.docx]

Supplementary Information

## Supplementary Methods: Stan Model

The full Stan model used for fitting the Beta mixture models is printed below.

//
// This Stan program defines a beta mixture model where both the mu and phi
// components are ordered such that each increasing component has a higher mu and
// lower phi.
// This version includes population-level effects and two hierarchical effects
//

data {
 int<lower=1> K; // number of mixture components
 int<lower=1> N; // number of data points
 real y[N]; // observations
 int<lower=1> J; // number of population level effects
 matrix[N,J] x; // matrix of population level predictors
 int<lower=1> L1; // number of groups within the hierarchical effect
 int<lower=1,upper=L1> ll1[N]; // vector matching observations to groups in hierarchical effect
 int<lower=1> L2; // number of groups within the hierarchical effect
 int<lower=1,upper=L2> ll2[N]; // vector matching observations to groups in hierarchical effect
}
parameters {
 simplex[K] theta; // mixing proportions
 ordered[K] intercept; // intercept of locations of mixture components
 positive_ordered[K] revphi; // scales of mixture components
 matrix[J, K] b; // covariate effects (different for each component)
 real<lower=0> sigma1[K]; // scale for hierarchical effect
 vector[L1] hier1_raw; // vector of hierarchical effect
 real<lower=0> sigma2[K]; // scale for hierarchical effect
 vector[L2] hier2_raw; // vector of hierarchical effect
}
transformed parameters {
 vector[K] phi = reverse(revphi); // use reverse ordering of phi for modelling
 matrix[K, N] invlogit_mu;
 // set up location of mixtures - inverse logit of sum of intercept and population
 // level effects
 for (k in 1:K){
 for (n in 1:N){
 invlogit_mu[k,n] = inv_logit(intercept[k] + x[n,] * b[,k] +
 sigma1[k]*hier1_raw[ll1[n]] +
 sigma2[k]*hier2_raw[ll2[n]]);
 }
 }
}
model {
 vector[K] log_theta = log(theta); // cache log calculation

 // priors
 phi ~ gamma(0.1, 0.1); // prior on scale parameters
 intercept ~ student_t(4, -1, 1); // prior on intercept
 to_vector(b) ~ normal(0,0.1); // prior on population level effects
 sigma1 ~ normal(0,0.1); // prior on hierarchical effect scale
 hier1_raw ~ normal(0,1);
 sigma2 ~ normal(0,0.1); // prior on hierarchical effect scale
 hier2_raw ~ normal(0,1);

 // model likelihood
 for (n in 1:N) {
 vector[K] lps = log_theta;
 for (k in 1:K){
 lps[k] += beta_lpdf(y[n] | invlogit_mu[k, n] * phi[k], (1 - invlogit_mu[k, n]) * phi[k]);
 }
 target += log_sum_exp(lps);
 }
}

## Supplementary Figures


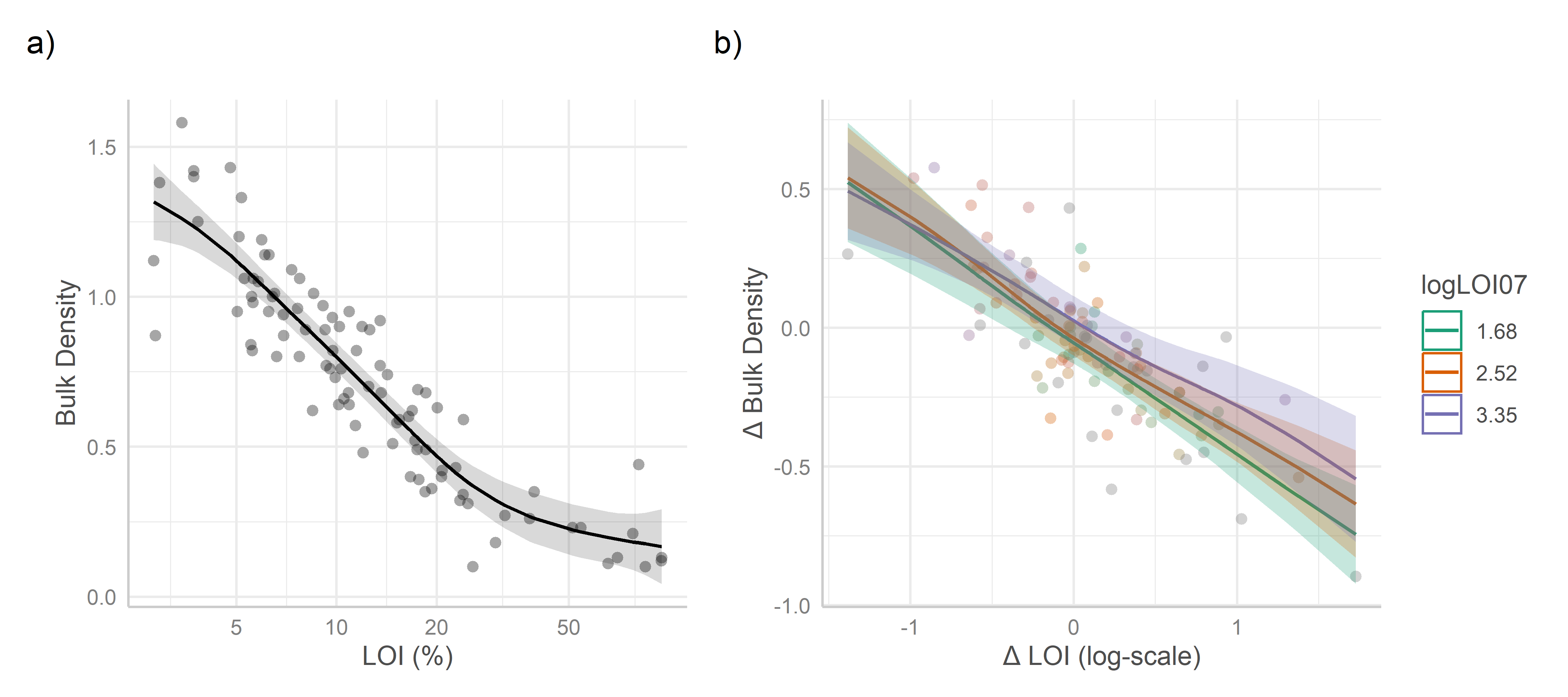


Figure S1: Data and model fits used to estimate bulk density as a function of LOI (a), and change in bulk density based on an interaction between change in LOI and the log of the original LOI (logLOI07) (b). The central line represents the mean estimate and the shaded ribbons the 95% CI. Model estimated change in bulk density (b) is shown for three example original LOI values, corresponding to ~5% (green), ~12% (orange), and ~29% (purple).


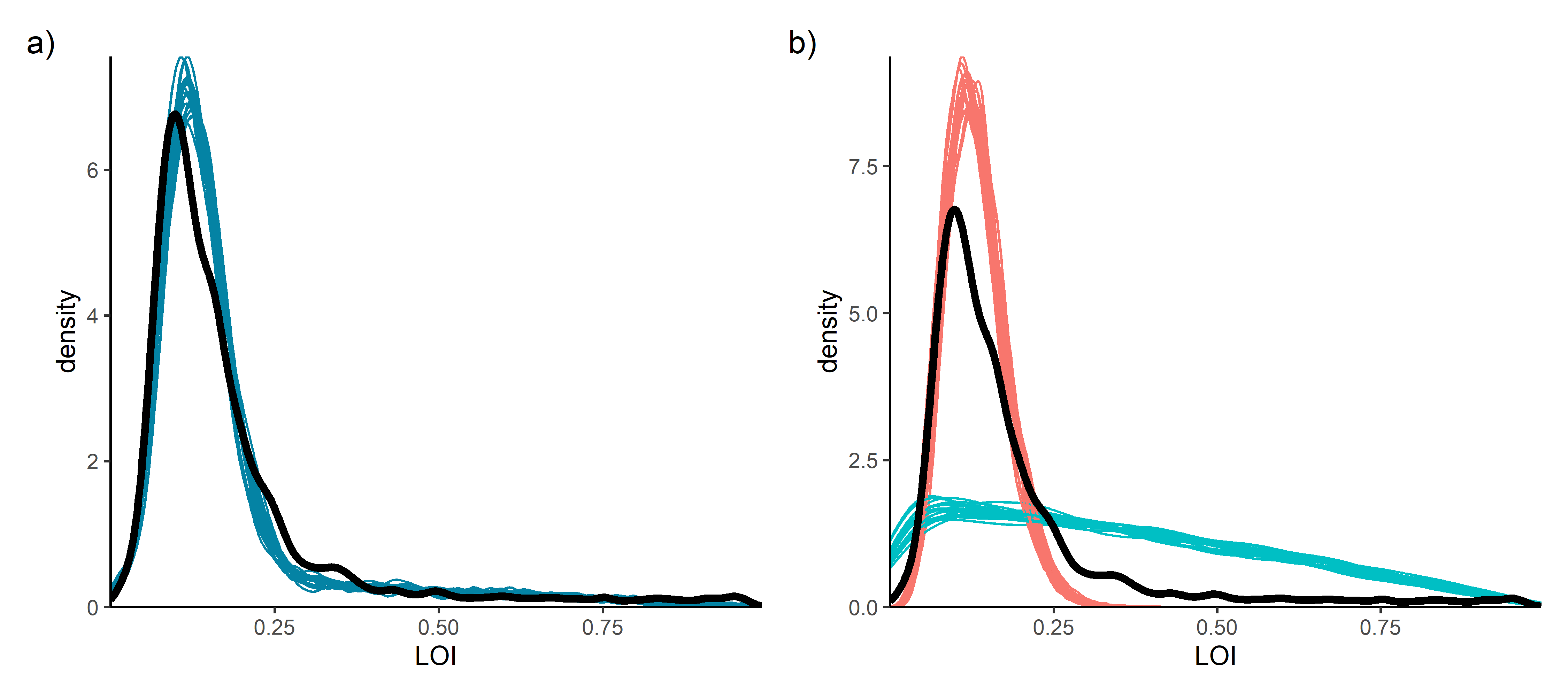


Figure S2: Density of actual data (black line) compared to model fit over all components (a, blue lines) and by mixture component (b). In (b) the model predicted densities are coloured by mixture components, with the high orange peak being the first mixture component and the lower blue lines the second mixture component.


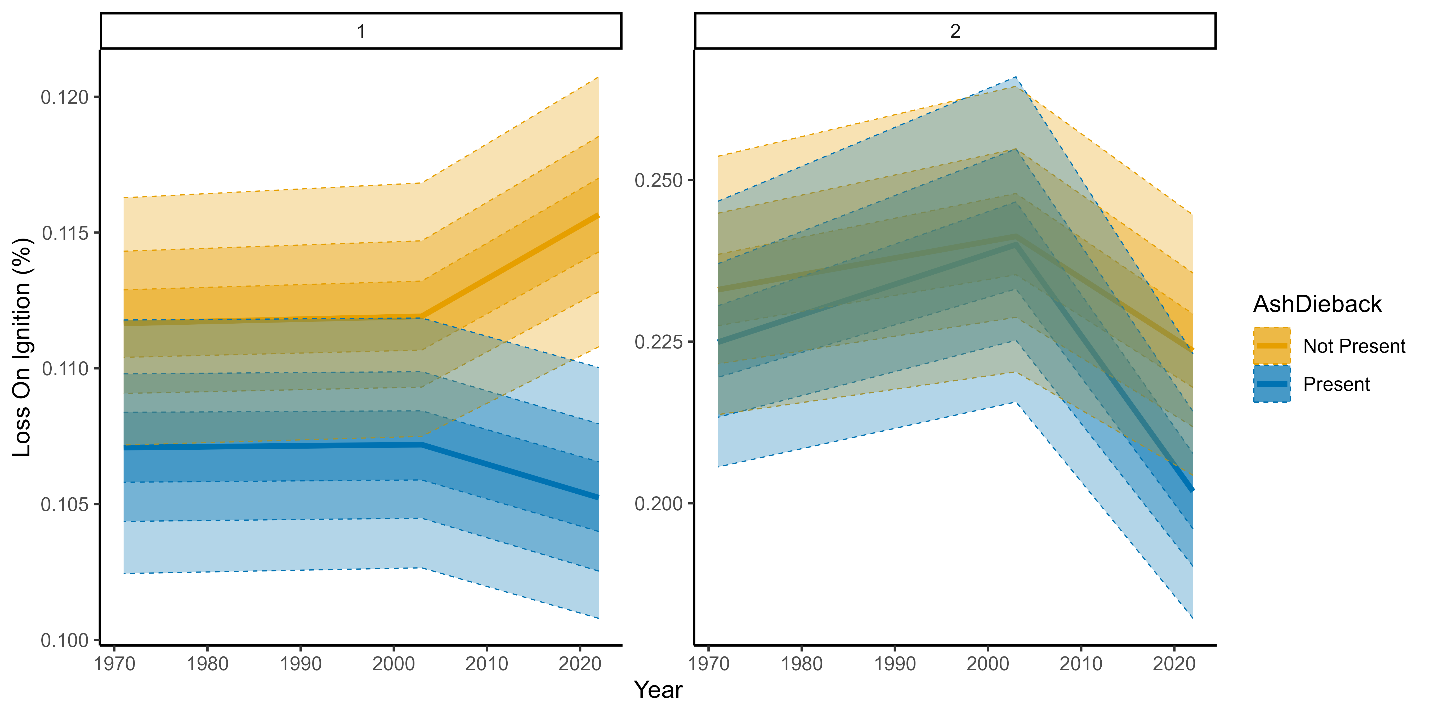


Figure S3: Change over time in ash plots with (blue) and without (yellow) ash dieback for the first mixture component (left, 80% of data, representing the majority of mineral soil measurements) and the second mixture component (right, 20% of data, representing the tail of high SOM values). The central line shows the median model estimated mean, while the shaded ribbons show the 25%, 50% and 75% confidence intervals of the estimated mean.

## Supplementary Tables

Table S1: Number of plots within each soil group according to the classification of Avery (1973).

| Major group description | Group description | Number of plots | Number of plots with ash | Number of plots with dieback |
| --- | --- | --- | --- | --- |
| Brown soils | Argillic brown earths | 87 | 32 | 18 |
|  | Brown alluvial soils | 12 | 7 | 5 |
|  | Brown calcareous earths | 98 | 60 | 33 |
|  | Brown calcareous sands | 6 | 6 | 3 |
|  | Brown earths (sensu stricto) | 504 | 176 | 74 |
|  | Brown sands | 154 | 44 | 24 |
|  | Paleo-argillic brown earths | 5 | 2 | 1 |
|  | **Total** | **866** | **327** | **158** |
| Ground-water gley soils | Alluvial gley soils | 17 | 11 | 7 |
|  | Argillic gley soils | 6 | 2 | 1 |
|  | Cambic gley soils | 35 | 11 | 7 |
|  | Humic gley soils (sensu stricto) | 4 | 2 | 1 |
|  | Humic-sandy gley soils | 2 | 0 | 0 |
|  | Sandy gley soils | 18 | 1 | 1 |
|  | **Total** | **82** | **27** | **16** |
| Lithomorphic soils | Pararendzinas | 3 | 2 | 0 |
|  | Rankers | 438 | 172 | 87 |
|  | Ranker-like alluvial soils | 2 | 1 | 0 |
|  | Rendzinas | 113 | 69 | 33 |
|  | Sand-pararendzinas | 1 | 0 | 0 |
|  | Sand-rankers | 64 | 12 | 8 |
|  | **Total** | **621** | **256** | **128** |
| Peat (organic) soils | Earthy peat soils | 4 | 0 | 0 |
|  | Raw peat soils | 13 | 2 | 1 |
|  | **Total** | **17** | **2** | **1** |
| Pelosols | Argillic pelosols | 1 | 0 | 0 |
|  | **Total** | **1** | **0** | **0** |
| Podzolic soils | Brown podzolic soils | 5 | 0 | 0 |
|  | Podzols (sensu stricto) | 13 | 2 | 2 |
|  | **Total** | **18** | **2** | **2** |
| Surface-water gley soils | Stagnogley soils | 34 | 17 | 6 |
|  | Stagnohumic gley soils | 9 | 1 | 0 |
|  | **Total** | **43** | **18** | **6** |
